# Thyroid cancer in Ecuador

**DOI:** 10.1186/s12885-020-07137-0

**Published:** 2020-07-09

**Authors:** Enrique López Gavilanez, Noemí Bautista Litardo, Manuel Navarro Chávez, Mario Hernández Bonilla, Angel Segale Bajaña

**Affiliations:** 1 AECE Research Group from Association of Clinical Endocrinologists of Ecuador, Guayaquil, Ecuador; 2 Servicio de Endocrinología, Hospital Docente de la Policía Nacional Guayaquil, No. 2, Avenida de la Américas S/N y E. Noboa, EC090150 Guayaquil, Ecuador

**Keywords:** Thyroid cancer, Mortality, Incidence, Ecuador

## Abstract

**Background:**

The incidence of thyroid cancer is increasing worldwide. This is not accompanied by a corresponding increase in mortality. In contrast, in most populations’ thyroid cancer mortality has been decreasing in recent decades, although there are some notable exceptions.

**Main body of the abstract:**

Relatively few studies focus on mortality and in Latin America we do not find evidence on the temporal trend of mortality. The study of the epidemiology of the thyroid cancer should be approached with a suitable methodology and with data based on the population. Trends should be expressed as an annual percentage of change and/or average annual rate of change. An appropriate method for analyzing trends in thyroid cancer mortality rates is the Joinpoint regression analysis. Previously published findings are described, and the methodology used is compared**.**

**Short conclusion:**

At the global level, Ecuador is one of the countries with the highest incidence rate of thyroid cancer. However, mortality data are scarce and not rigorous. It is important to raise awareness of updated and reliable population-based data on the trend of thyroid cancer mortality in Eccuador.

## Background

The incidence of thyroid cancer (TC) has been found to be up to ten times higher in developing countries than in developed countries, and TC mortality has usually shown unchanging or declining rates in both types of countries [1, 2]. However, recently, the mortality rate for TC has increased in several countries, including the United States [3].

## Main text

We have read with great interest the study of Salazar-Vega et al., titled “Thyroid Cancer in Ecuador, a 16 years population-based analysis (2001–2016),” which was published on April 2, 2019 in the BMC Cancer [4]. In this study, the authors declare that “In Ecuador there is no reports available of the epidemiology of this type of cancer,” and they also affirm that “… the majority of Latin-American countries such as Ecuador, Perú, Bolivia or Paraguay have not reported TC trends, incidences or any epidemiological data in the last decade” [4]. However, some previous publications have addressed several aspects of the epidemiology of TC in Ecuador [3, 5–10]. In 2018, two publications in indexed journals analyzed the epidemiology of TC, one of a national and the other of a regional nature. In August 2018, Lopez et al., published a time series study that analyzed the trend of the mortality rate for TC in Ecuador over a period of 26 years, from 1990 to 2016 [5]. This study included numerous events (2107 deaths), covered a long period (26 years), and involved the entire Ecuadorian population. Their findings indicate a persistent increase in TC mortality in women between 1997 and 2016 and a moderate decrease in TC mortality in men from 1990 to 2016 [5]. In addition, Corral et al., in 2018, published a study of trends in cancer incidence and mortality, including TC over three decades in Quito, Ecuador. In the analysis of the time trends, the Joinpoint regression was used [6]. Lastly, the bulletin epidemiological No. 8 of Cancer Fighting Society (SOLCA) Guayaquil Nucleus describes mortalities due to TC in Guayaquil, Ecuador, from 2008 to 2017 [7].

In another part of his paper, Salazar-Vega et al. [4] declare that “All the data was obtained from the official records reported by the Ministry of Public Health’s and retrieved from the public databases of the Vital Statistics Deaths and Births Databases and the National Institute of Census and Statistics (INEC)” [11]. A limitation of hospital discharge is that it is not designed to adequately discriminate between the multiple occasions on which the same patient enters and leaves a hospital or for cancer cases that do not require hospitalization [6]; therefore, there is a high risk of duplication of cases, which constitutes an important statistical bias, as it would override the calculation of the incidence and years lived with disability (YLD). On the contrary, this database registers deaths from TC as the basic cause of death in each year; if it allows making to estimate the annual mortality rate and the trend of this in periods long time.

Salazar-Vega et al. [4] describe in “Trend” the increase in frequency per year in mortality rate, as they state that “…. during the same time TC mortality increased from 0.48 to 0.87 per 100,000 individuals in the overall 16 years period,” but this does not, in any way, represent a measure of the tendency of TC mortality. In August 2018, Lopez et al. explored the trend of TC mortality in Ecuador [5]. To assess temporal changes in TC mortality, the mortality rates were estimated yearly from 1990 to 2016. Age-related mortality rates were standardized by the direct method using the world population (based on the World Health Organization data) as the standard. Joinpoint regression analysis was used to estimate trends in age-standardized mortality rates for both sexes. In addition, mortality rates were predicted for 2030, 2040, and 2050. The trends were expressed as an annual percentage of change (APC) and average annual percentage of change (AAPC). The trends in the mortality rates were modeled through Joinpoint regression analysis. A final best-fit model was selected with the estimated APC based on a trend within each segment. AAPC estimation involves using the underlying Joinpoint model to calculate a summarized measure over a pre-specified fixed interval. This allows us to use a single number to describe the average of APCs over several years. It is valid even if the Joinpoint model indicates changes in trends during those years. It is computed as a weighted average of the APCs of the Joinpoint model with weights equal to the length of the APC interval. This calculation was made with the software provided by the National Cancer Institute, which is freely available and a correct way to measure the tendency of different cancer types (Jointpoint Regression Program, Statistical Methodology and Applications Branch, Surveillance Research Program, National Cancer Institute. Version 4.5.0.1. 2017).

At the global level, because TC incidence showed a sharp increase in the early 1990s, a noticeable increase in the death rate should have been perceived after 10–20 years. In fact, the trend in TC mortality, as observed by the The Surveillance, Epidemiology, and End Results (SEER) Program from 2001 to 2010, indicates a total AAPC of 0.9% (0.9% in women and 1.6% in men) [12]. Consequently, TC mortality would continue to increase in relation to the increase in high incidence, albeit with a delay, which would be justified by the low aggressiveness of TC [13]. In the paper of Lopez et al. [5], it is observed that the increasing mortality rate in women follows a pattern resembling that reported in the United States, Australia, and United Kingdom, with a decrease observed till the end of the 1990s, followed by stabilization or subsequent increase [3]. TC mortality in Ecuador tended to decrease in men and women from 1990 to 1998. Although this trend persisted for men, TC mortality in women increased from 1998 to 2016. In addition, the TC mortality rate is predicted to increase for both sexes in the coming decades. The precise reason for the observed increase in TC mortality in Ecuadorian women from 1998 onwards remains unclear [5].

## Conclusion

At the global level, Ecuador is one of the countries with the highest incidence rate of thyroid cancer. However, mortality data are scarce and not rigorous. It is important to raise awareness of updated and reliable population-based data on the trend of thyroid cancer mortality.

## Data Availability

All the information used for this analysis can be found in the following. website http://www.ecuadorencifras.gob.ec/estadisticas-de-camas-y-egresoshospitalarios-bases-de-datos/ and contains a full set of records in a yearly manner from 1990 to 2016.

## Abbreviations

TC: Thyroid cancer
APC: Annual percentage of change
AAPC: Average annual percentage of change
YLD: Years lived with disability
